# X-ray transfocators: focusing devices based on compound refractive lenses

**DOI:** 10.1107/S0909049510044365

**Published:** 2010-12-21

**Authors:** Gavin B. M. Vaughan, Jonathan P. Wright, Aleksei Bytchkov, Michel Rossat, Henri Gleyzolle, Irina Snigireva, Anatoly Snigirev

**Affiliations:** a European Synchrotron Radiation Facility, 38043 Grenoble Cedex, France

**Keywords:** compound refractive lens, X-ray optics, monochromator, microfocus

## Abstract

This paper describes a tunable X-ray focusing apparatus, referred to as a transfocator, based on compound refractive lenses. By varying the number of lenses in the beam, the X-ray energy focused and the focal length can be varied continuously throughout a large range of energies and distances. The instrument can be used in both white and monochromatic beams to focus, pre-focus or collimate the beam. The transfocator can be used with other monochromators and/or other focusing elements, leading to significant increases in flux. Furthermore, the chromatic nature of the focusing means the transfocator suppresses harmonics and can also be used as an extremely high flux broad-band-pass monochromator. These devices have been installed in the first optics and second experimental hutches at the ID11 beamline at the ESRF.

## Abstract 2

A tunable X-ray focusing and/or monochromating device, called a transfocator, is described. Examples of its implementation on ID11 at the ESRF are given.

## 1. Introduction

A large variety of X-ray focusing elements are now available, such as single and double Kirkpatrick–Baez (KB) geometry (Kirkpatrick & Baez, 1948 ▶; Liu *et al.*, 2005 ▶; Hignette *et al.*, 2007 ▶; Mimura *et al.*, 2009 ▶) normal and multilayer mirrors, bent crystals (Lienert *et al.*, 1998 ▶; Suortti & Schulze, 1995 ▶; Suortti *et al.*, 2001 ▶) and zone plates (Baez, 1961 ▶). Each type of optics has advantages and inconveniences. Mirror and crystals offer excellent efficiency and may be used to produce intense small beams, but their reflective or diffractive nature means that they multiply angular instabilities. Zone plates are in-line optics and can be used to produce extremely small beams, but are compromised with respect to background and are inefficient at high energy, although it is possible to stack them to partially overcome the poor efficiency (Shastri *et al.*, 2001 ▶; Maser *et al.*, 2002 ▶; Kamijo *et al.*, 2003 ▶; Snigireva *et al.*, 2007*a* ▶,*b* ▶).

Since their development a little over 12 years ago (Snigirev *et al.*, 1996 ▶), the use of X-ray refractive lenses has rapidly expanded (Lengeler *et al.*, 1999*a* ▶,*b* ▶; Kohn *et al.*, 2003 ▶; Snigirev & Snigireva, 2008 ▶) and they are now in common use on synchrotron beamlines. Compared with other focusing elements, refractive lenses present several attractive features, being simple to align and relatively insensitive to misorientations. Since refractive lenses are in-line optics, they are more stable with respect to angular vibrations in comparison with deflecting optics. They can be adapted to very high X-ray energies by modifying their composition and number, and can be inserted and removed from the beam to allow fast switching of the beam size. As the index of refraction for refractive lenses is extremely close to unity and energy dependent, a substantial well defined number of lenses are necessary to focus X-rays of a given energy at a given distance. For this reason, systems with a tunable number of lenses have been proposed (Snigireva *et al.*, 2004 ▶; Snigirev *et al.*, 2007 ▶) to provide permanent energy and focal length tunability. These ‘transfocators’ are comprised of several cartridges containing different numbers of lenses, such that the focal distance can be continuously adjusted by insertion or retraction of one or more of the lens cartridges.

An in-air transfocator (IAT), first described by Snigirev *et al.* (2009*a* ▶), has been in operation at the ID11 beamline at the European Synchrotron Radiation Facility (ESRF) for almost two years. Based on the success of the IAT, an in-vacuum transfocator (IVT) has now been built and installed at ID11. The IVT benefits from being closer to the X-ray source (31.5 m) where it captures a larger proportion of the diverging X-ray beam. Unlike the IAT, the IVT is water-cooled to allow use in the white beam. ID11 has recently been extended to be a ∼100 m long-beamline (Vaughan *et al.*, 2011 ▶), one of the pilot projects for the ESRF upgrade. The variable focal length of the transfocator focused beam means that it can be exploited at all of the beamline experimental stations. At 94 m from the source, flux gains of the order of 5 × 10^4^ are achieved with respect to an unfocused beam. The gain in the microfocused beam at 42 m from the source is even more substantial, over 10^5^.

The IVT is very flexible and has been used in several different configurations as a stand-alone focusing device in the monochromatic beam, giving vertical spot sizes ranging from 6 to 42 µm depending on the focal distance. Also the IVT can act as a pre-focusing device to be used in conjunction with a downstream microfocusing or nanofocusing element, leading to enormous flux gains. Finally, without any other optics, the IVT acts as a *longitudinally dispersive monochromator*, producing beams with about 1% band pass and several micrometers vertical size.

For microfocusing and nanofocusing applications at long (∼100 m) focal length, the small beam size from the IVT corresponds well with the acceptance of downstream focusing optics. The pre-focused beam from the IVT has been used with subsequent focusing from further refractive lenses or a silicon nanolens chip (Snigirev & Snigireva, 2008 ▶; Snigirev *et al.*, 2009*b* ▶), or a multilayer system. The flux gain is substantial, with only slight degradation in the spot size. For example, focusing the IVT behind the focal point of a series of short-focal-length lenses produces a factor of 30 gain in the focal spot, with only a 75% increase in spot size. An even larger flux gain is found when using the nanofocusing chip, a factor of 40, with the spot being broadened from about 200 to 450 nm vertically at 50 keV.

As a single optical device in the white beam, the IVT can act as a fundamentally new kind of monochromator, delivering impressive flux in a ∼1% band-pass beam (a factor of 75 with respect to the ∼10^−3^ band-pass beam when used in conjunction with a double Laue monochromator). The energy selectivity of the IVT comes from the chromatic nature of the focusing; with a relatively short focal length the depth of focus is sufficiently short that only a narrow band pass is focused at a given longitudinal point. At the focal point, this leads to a two order of magnitude increase in the flux at this selected energy with respect to the rest of the spectrum. The band pass of this monochromator is well matched to exploit the spectrum of the harmonic peaks of an undulator insertion device at a third-generation storage ring.

## 2. Mechanical design and construction

### 2.1. Construction and implementation

The mechanical design of the transfocator is based on that of the in-air prototype described earlier (Snigirev *et al.*, 2009*a* ▶), which has been in use at ID11 for microfocusing applications for two years. In the present case the design has been upgraded in order to function in high vacuum near the front of the beamline, and cooled to accept white beam.

**Figure 1:**
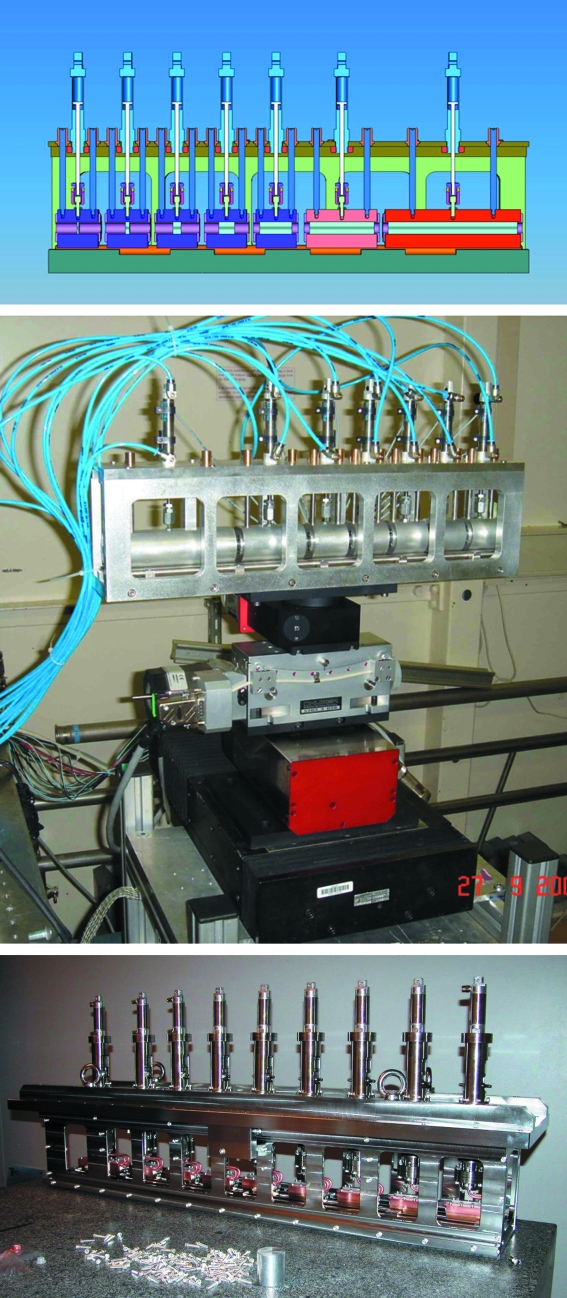
Schematic illustrating the transfocator concept (top), the IAT installed on the beamline (middle) and the IVT during assembly (bottom).

Both transfocators are based on a system of pneumatically actuated cartridges containing a geometric progression of numbers of lenses, allowing, in the case of the IAT, between two and 254 Al lenses (in steps of powers of two) and between one and 63 Be lenses and 32 to 96 Al lenses for the IVT. The designs of the two transfocators are shown in Fig. 1 ▶.

The individual lenses are made from polycrystalline aluminium or beryllium by a pressing technique (Lengeler *et al.*, 1999*a* ▶; Tummler, 2000 ▶). The aperture of each lens is 1 mm; the web thickness is around 50 µm. The lens radius of curvature is 200 µm. The paraboloids are held and centered in a brass (for Al) or covar (for Be) frame of diameter 12 mm and thickness 1 mm (for Al) and 2 mm (for Be).

The IAT was manufactured based on an in-house design. Pneumatic actuators are used to move cartridges in and out of the beam independently, changing the number of lenses from two to 254 with steps of two lenses. Pneumatic actuators (Festo reference DSNU-10) with a stroke of 20 mm are driven by bi-stable solenoid valve (WIT). The lens cartridges are made of aluminium and have 12 mm (10 µm tolerance) internal and 40 mm (12 µm tolerance) external diameter.

A critical design parameter is the straightness of the rails upon which the cartridges rest. By carefully aligning the cartridges with respect to one another, it is sufficient for the instrument to be mounted on a single goniometer that controls the position perpendicular to the beam and the tilts to ensure the lenses are parallel to the beam. The IAT has additionally a 1.7 m translation along the beam direction, in order to tune the focal length.

Based on the design of the IAT, the IVT is considerably more complex from an engineering viewpoint, as the cartridges must translate in vacuum, must be water cooled, and the entire chamber is considerably larger. The apparatus was designed in-house and manufactured by Cinel Strumenti Scientifici S.r.l. in Padova, Italy. The high quality of manufacture is evident in the perfect alignment of all nine cartridges over a length of more than 1.5 m. The IVT is mounted on a reconstituted marble support and permanently attached using epoxy following initial alignment, in order to provide the highest possible rigidity with respect to vibrations.

All moving parts near the IVT (anywhere in the entire optics hutch) have been eliminated in order to reduce the vibrations. Cooling of the IVT is achieved by a gravity flow system intended to circulate the cooling liquid (water and ethylene glycol) without introducing any vibrations. This configuration is highly robust against vibrations, allowing the imaging of an unperturbed X-ray source.

### 2.2. Optical design

The short focal distance of the IAT means that many lenses are required for focusing, and that a smaller focal spot can be achieved. The tunability of the IAT allows submicrometer focusing up to 47 keV. The IVT is located further from the sample and thus requires significantly fewer lenses, and means that the depth of focus (DOF) is larger, allowing more freedom in focal length.

**Figure 2:**
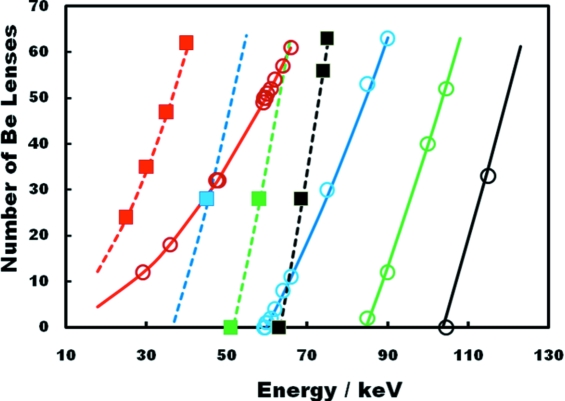
Theoretical (lines) and measured number (points) of Be lenses needed to optimize focus at 95 m. The different curves correspond to coupling the Be lenses with 0 (red), 32 (blue), 64 (green) or 96 (black) Al lenses. The closed squares refer to a focus at 42 m, the open circles to a focus at 94 m.

**Figure 3:**
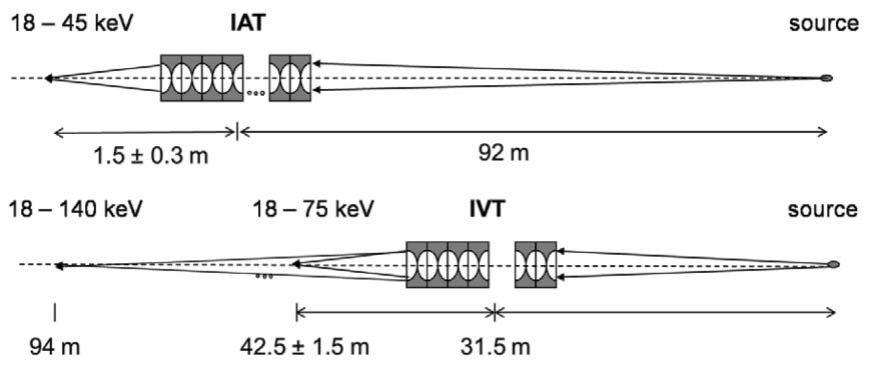
Implantation of the IAT (top) and IVT (bottom). The IAT is configured to give a ∼1:15 focusing of the source, whereas the IVT operates in either a 3:1 or 1:2 configuration. Other beamline optics are not shown.

The IAT has seven pneumatically actuated cylindrical cartridges containing 2, 4, 8, 16, 32, 64 and 128 Al lenses, giving tunability up to 47 keV. At the emplacement 92.5 m from the source the focal distance is ∼1.5 m and can be adjusted *via* a motorized translation of the lenses along the beam. The IVT consists of nine water-cooled cartridges containing 1, 2, 4, 8, 16 and 32 Be lenses, and 32 and 64 Al lenses. The combination of these cartridges allows complete tunability between 18 and 125 keV at 94 m, and 18 to 75 keV at 42 m. The number of lenses necessary to optimize the focus at a given distance is in perfect agreement with theory (Lengeler *et al.*, 1999*a* ▶; Kohn *et al.*, 2003 ▶), and follows smooth curves as shown in Fig. 2 ▶. A very simple approximation can be used to compute the lens requirements: one Be lens has a focal length in meters given by *kE* ^2^, where *E* is the X-ray energy in keV and *k* is 0.296 m keV^−2^. An aluminium lens has a focal length equivalent to ∼1.568 Be lenses. The number of lenses required at ID11 can then be computed simply *via*
               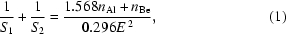
where *S*_1_ and *S*_2_ are the object and calculated image distances, respectively, as shown in Fig. 3 ▶. We find that the effective number of lenses is given by 1.568*n*_Al_ + *n*_Be_ equal to 0.0376*E* ^2^ at 42 m distance from the source and by 0.014*E* ^2^ at 96 m, where *E* is in keV and *S*_1_ and *S*_2_ are in meters. Using this approximation, all of the fits in Fig. 2 ▶ can be produced using only two free parameters.

At low to modest energies, Be lenses are preferable owing to their low absorption. The ratio of Be to Al lenses mounted in the IVT was selected to optimize the efficiency of the device. The crossover in efficiency between Al and Be lenses owing to their relative absorption and scattering power takes place at approximately 65 keV, where all 63 Be lenses are needed to focus at the primary end-station at 94 m. Therefore the instrument is optimized for long-focal-length operation throughout the current energy range of ID11, 18–125 keV. The 63 Be lenses correspond in focusing power to ∼40 Al lenses; it is therefore only necessary to be able to vary the number of Al lenses by groups of less than 40. Two cartridges with 32 and 64 Al lenses have been implemented, allowing for some overlap between Be/Al combinations.

For the secondary experimental station at 41–44 m from the source the IVT can be used to microfocus in the range ∼25–75 keV, producing spots of the order of 6 µm × 45 µm. It can also be used as a condenser for post focalization. The two experimental configurations are shown schematically in Fig. 3 ▶.

## 3. Performance

### 3.1. Performance of the IVT in monochromatic beam at 94 m

The IVT was tested in the monochromatic beam throughout its operational range at the 94 m end-station. The monochromatic beam was provided by a horizontal bent Laue–Laue monochromator (Baruzzo *et al.*, 2008 ▶). Owing to the moderate to high energies used and the horizontal Laue geometry the heat load on the monochromator is small, and the beam is extremely stable with respect to vibrations and thermal fluctuations, meaning that there is only negligible vertical source size broadening (Vaughan, 2010 ▶). Band pass from the monochromator is in the 1 × 10^−3^–10 × 10^−3^ region, depending on energy and the section of the variable-thickness crystals used (Vaughan, 2010 ▶).

The number of lenses necessary for a given distance and energy were calculated from equation (1). Several regions of overlap exist in which two different solutions of lens combinations exist, *e.g.* from ∼60 to 66 keV, where either only Be lenses or a combination of 32 Al and 0–11 Be lenses can be used. In this case it is slightly preferable in terms of flux (there is no difference in focal size) to use all Be. In higher energy overlap regions it is preferable to use Al lenses as early as possible, although in both cases the effect is relatively small.

The transmission, defined as the ratio of the input to the output beam, varies from 30 to 70%. In this case the real aperture is larger than the incoming beam, which is limited vertically by the intrinsic beam size from the undulator, and horizontally by a collimating aperture upstream from the IVT. From the transmission and measured spot size we can calculate the gain, defined as the increase in flux density in the focal spot with respect to slitting the unfocused beam down to the same dimensions. Vertical spot sizes of ∼40 to 60 µm were found throughout the energy range, with very little variation. Horizontal spots were closer to 300 µm, broadened by dispersion from the horizontal geometry of the monochromator. The beam at 95 m was taken to be 1 mm × 2.4 mm, and the point spread function of the camera system used to measure the focused beam was taken to be five pixels and Gaussian. The real point spread function is possibly larger and somewhat energy dependent. Thus the gain of the system is of the order of 100 to 200.

As the focal length is quite long, the DOF at the focal spot is also substantial. If we consider the physical aperture of the Be lenses (0.85 mm) to be the optical aperture, we would expect a vertical DOF of over 6 m for a 44 µm spot at 95 m. The vertical beam at the transfocator is actually smaller than the vertical aperture (varying with energy and undulator setting) but even the smallest beams should give a DOF of several meters, sufficient to focus at the sample (or other desired) position.

**Figure 4:**
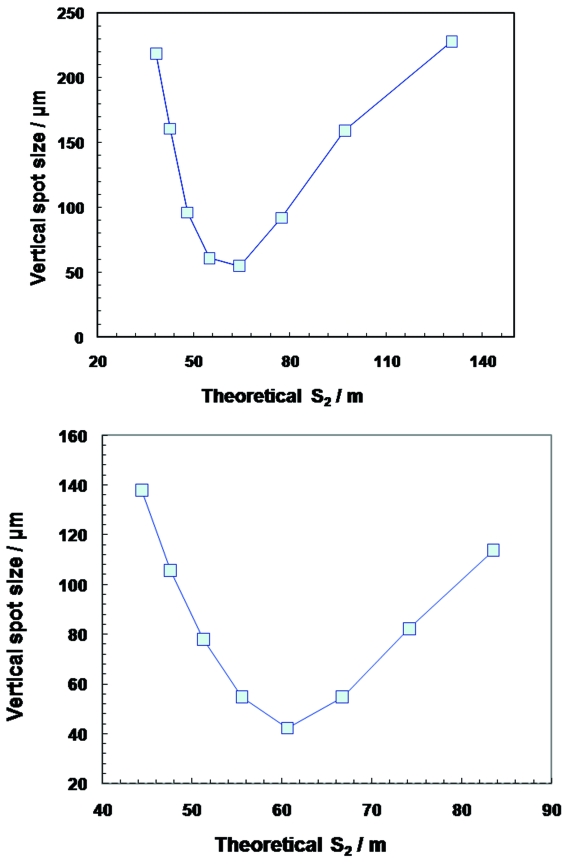
Theoretical *S*_2_ values for a given number of lenses *versus* the measured vertical spot size at 62.5 m from the IVT (94 m from the source) for 36 keV (top) and 47.5 keV (bottom). Each point corresponds to adding an additional Be lens.

The tunability of the transfocator is thus sufficient at this distance even with a fixed focal length, down to the lowest energies used by ID11, although usage at lower energy or shorter focal distance requires selection of the energy matched to the number of lenses in order to place the sample position within the DOF. Fig. 4 ▶ shows the optimization of the number of lenses at 36 and 47.5 keV. At the lower energy, insertion of a single additional lens displaces the focal spot by about 9 m near the focal position, larger than the DOF. Particular energies, matched to the focal length, must therefore be used. At 47.5 keV, insertion of a single lens moves the focal spot near the focus by about 6 m, comparable with the DOF.

### 3.2. Use with monochromatic beam at 42 m

At the 41–44 m end-station, the transfocator focuses to ∼6 µm vertically between ∼25 and 75 keV. At this distance, the DOF is only ∼60 mm, making it necessary to translate the sample and detector along the beam in order to position the focal spot as desired. The experimental station is conceived for this application. The focusing of the IVT was checked at 35.61 and 71.5 keV; the measured spot size and DOF agree with the calculations.

**Figure 5:**
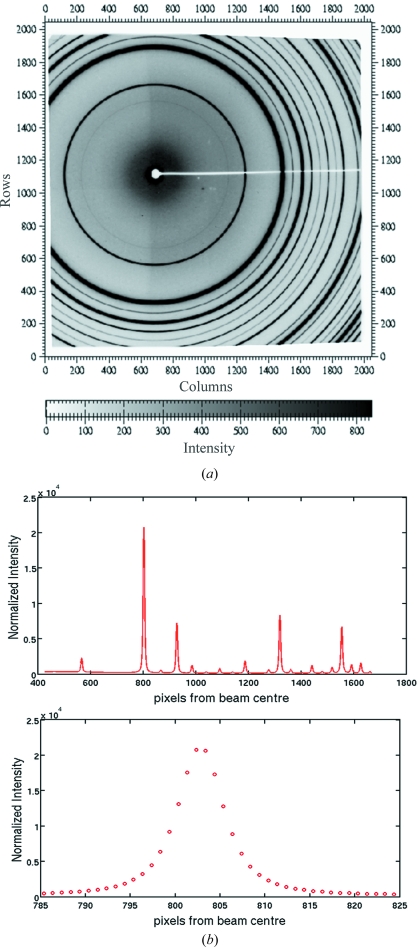
Diffraction pattern of Yb_2_O_3_ taken with IVT at 42 m and 35.61 keV monochromatic beam (*a*) and the azimuthally integrated equivalent diffractogram (*b*).

In order to confirm that no unexpected aberrations are introduced into the beam which would adversely affect the quality of diffraction data, diffraction patterns of Yb_2_O_3_ were collected in this configuration. As shown in Fig. 5 ▶, the data do not show any evidence of optical aberrations, with peak shapes perfectly symmetrical.

### 3.3. Use of the IVT in white beam

#### 3.3.1. As a focusing monochromator

**Figure 6:**
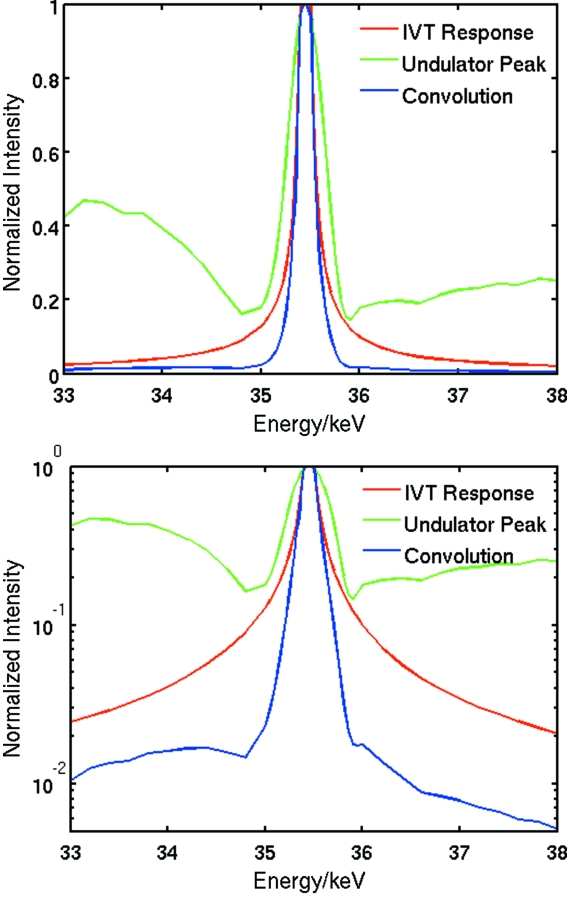
Theoretical energy spectrum in a 5 µm high beam at the focal position for 48 Be lenses, from a theoretical constant flux *versus* energy source (red), a measured undulator peak at the same energy (green) and the convolution of the two (blue). FWHM is about 0.4% and P/B about 40 for the IVT response alone, and 0.6% and 100 for the convolution.

The IVT can be used without any other optics as a *longitudinally dispersive focusing monochromator*, with a band pass proportional to the focal length. As the focusing of the lenses is chromatic, only a single energy is focused at a given distance. Shorter focal lengths give a smaller DOF and therefore energies will be more highly localized longitudinally as focal length decreases. Fig. 6 ▶ shows theoretical spectra at *S*_2_ = 10 m with 48 Be lenses used, assuming a constant flux/energy source.

A super-Lorentzian energy profile is observed in the simulation, with a peak-to-background (P/B) ratio of ∼40, and a full width at half-maximum (FWHM) of 218 eV or ∼0.61% band pass. The large tails, however, lead to a large integral breadth (β) of 361 eV or 1.0% {β ≡ πΓ/2 [η + (1 − η)/ln 2], where Γ is the FWHM and η is the pseudo-Voigt mixing parameter}.

The peak flux, width and the P/B ratio can be improved by tuning the undulators to the same energy as that of the optimized focus. Fig. 6 ▶ shows the theoretical effect of tuning the U22 in-vacuum undulator (Chavanne *et al.*, 2003 ▶) to a sharp odd peak. The total flux is improved substantially, and the P/B ratio is more than doubled to 100. The intrinsic form and width of the undulator peak renders the resultant peak somewhat asymmetric, but substantially lowers the tails of the peak, as can be seen in Figs. 6 ▶ and 7 ▶. The FWHM of the pseudo-Voigt fit is now 198 eV (0.56%) and β = 273 eV (0.77%).

In such a configuration, the band pass will scale with the focal spot size/focal length, as shorter focal lengths give a smaller DOF and therefore energies will be more highly localized longitudinally. The focal length to the experimental station at 42 m allows a good match between the band pass of the IVT and that of an undulator peak. At this distance the DOF is a few millimeters, allowing thick samples to be studied without introducing an energy gradient over the thickness of the sample.

**Figure 8:**
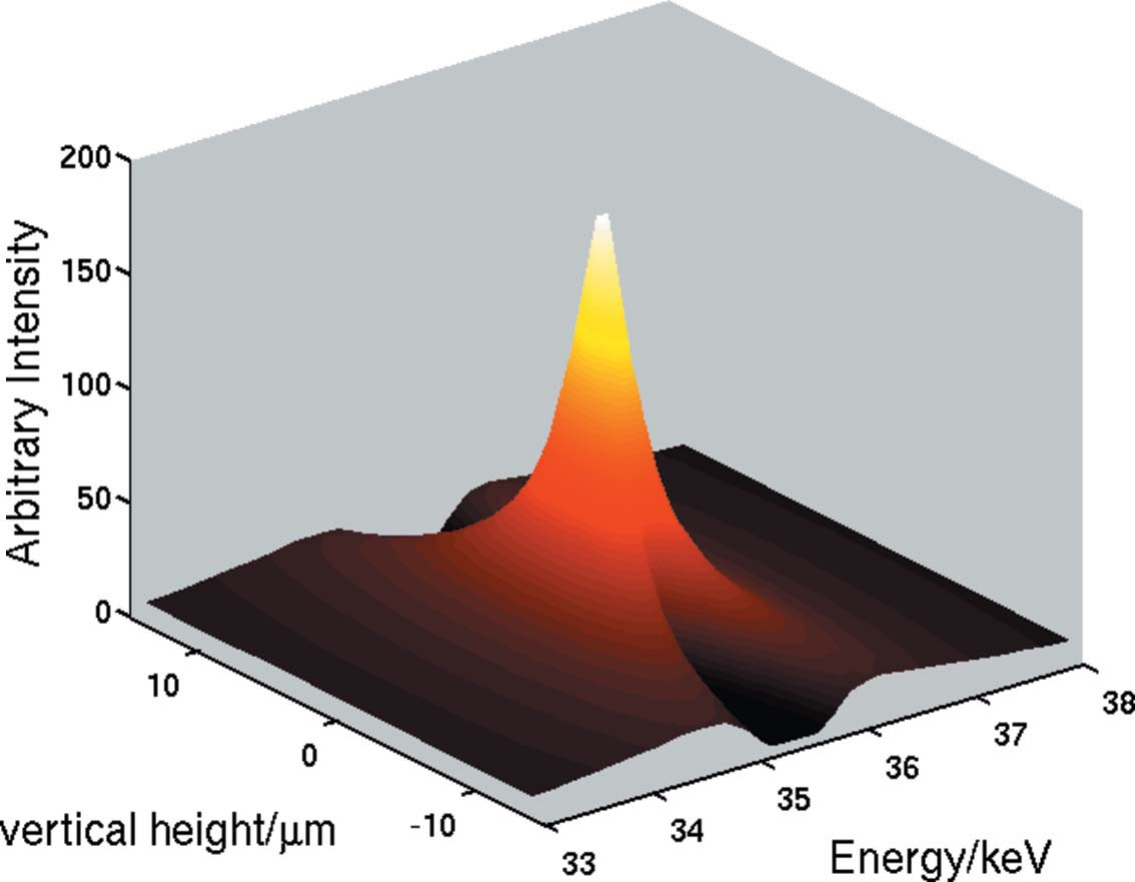
Theoretical vertical and energetic distribution of the flux at the focal length for 35.46 keV. The DOF is about 6 mm.

The curves above were calculated at the focal point, integrating over the focal spot perpendicular to the beam. Fig. 8 ▶ shows the (theoretical) distribution in space (vertically) and energy of the beam at the focal length, which in this case has a depth of some 6 mm. It can be seen that, even without any slitting, the spot is well defined in both height and energy.

**Figure 9:**
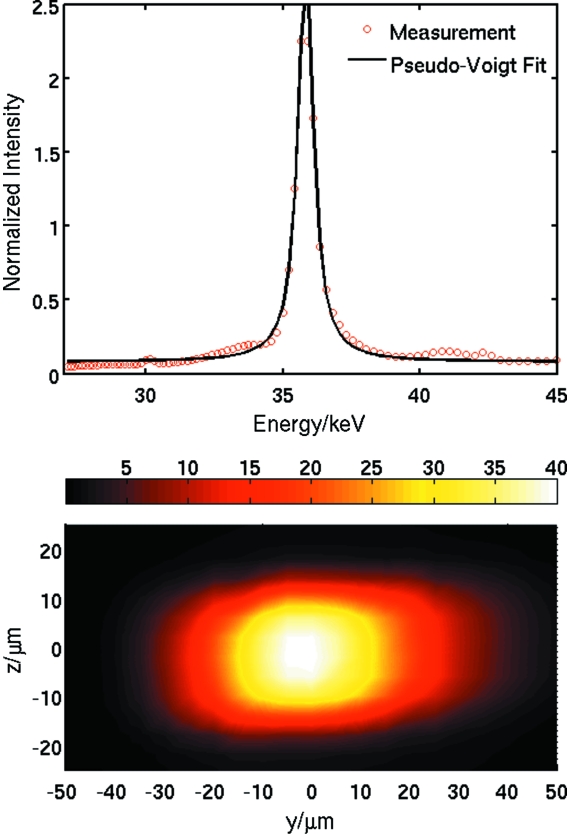
Top: measured spectrum through a 10 µm × 10 µm pinhole of the beam optimized for 35.5 keV. Bottom: spatial distribution of the flux at 35.500 ± 0.005 keV perpendicular to the beam at the focus.

In order to test these simulations, the IVT was aligned for 35.61 keV at about 10 m focal length. The number of lenses and undulator peak were optimized with the monochromator present. The monochromator was then removed, and the energy and spatial distribution of the beam was measured by scanning a small (10 µm × 10 µm) pinhole perpendicular to the beam at the focal spot. At each position a downstream analyzer crystal was scanned to record the energy spectrum over a large range. Fig. 9 ▶ (top) shows the result of such as scan with the slit positioned at the flux maximum. The beam has a FWHM of about 337 eV, or just less than 1%. The background shows some features associated with the undulator spectrum, but the signal-to-noise ratio is about 40. The flux increases by a factor of 75 with respect to the monochromated beam, whereas the band pass increases by a factor of less than ten.

Fig. 9 ▶ (bottom) shows the spatial distribution of the peak energy (broadened by the 10 µm × 10 µm slit) measured by scanning the slit with the analyzer at fixed angle; the peak is less than 10 µm vertically, whilst the horizontal broadening is caused by the monochromator geometry.

Such ultra-intense, stable and focused moderate band-pass beams can be useful for several applications in which high energy resolution is not necessary, such as scattering from liquids or poorly crystalline materials. A similar scheme, using alligator lenses, was proposed by Jark (2002 ▶).

#### 3.3.2. IVT in white beam with a secondary monochromator and/or focusing optics

The IVT can be used as a white-beam condenser/collimator for subsequent focalization and/or monochromatization with other optical elements. This has been demonstrated by use in conjunction with a KB multi-layer mirror focusing device. The KB is installed at approximately 92 m from the source and 1.5 m before the final experimental station. This system is used to focus X-rays over the entire range of energies used at ID11, but generally above 50 keV, and also acts as a broad-band-pass monochromator if used without other monochromating devices.

When used at high energy, the incident angle is very small, although the maximum feasible *d*-spacing, ∼20 Å, has been used for the multilayers. The footprint of the beam is thus large, necessitating long and consequently less perfect mirrors for large acceptance. Mirror sag and multilayer imperfection lead to a degradation of the focused beam and difficulties in aligning the system. Therefore the incident beam is generally reduced in size by slits before the KB system, resulting in significant loss of flux. By using the IVT as a condenser, the footprint of the beam covers a much smaller area on the mirror, which can be perfectly bent and which has greater stability than the entire length of the mirror.

This configuration was tested using 100 keV beam. The IVT was first aligned with 64 Al and 40 Be lenses, giving a beam size measured at the sample position on the high-resolution camera of about 50 µm × 300 µm. The KB was then aligned in the beam and the horizontal and vertical focuses were optimized by bending the mirrors. After these procedures we obtained a homogeneous 8 µm × 9 µm beam (the smallest beams produced by the KB system at this energy are 5 µm × 5 µm) at a sample position 1.4 m away from the second mirror. The thermal stability was measured with overnight stability tests. The beam size and positions remained stable to better than 1 µm with no additional feedback

### 3.4. Compound transfocator

#### 3.4.1. Performance of the IAT

The IAT, with 0–254 Al lenses, has been in operation for two years for microfocusing applications (Snigirev *et al.*, 2009*a* ▶). It is located at 92.5 ± 0.2 m from the source, and has a working length to the sample *S*_2_ of 1.3–1.7 m, allowing focusing up to ∼47 keV. In this configuration vertical spot sizes down to 800 nm have been measured.

**Figure 10:**
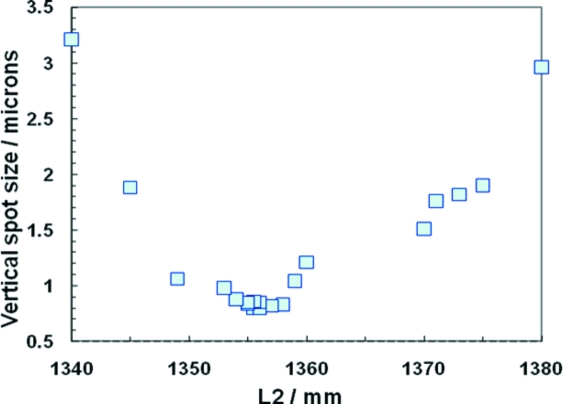
Optimization of the focal spot of the IAT, with 128 lenses at 29.6 keV.

The data in Fig. 10 ▶ were measured by scanning a knife edge through the beam at the focal spot. Owing to the short focal length, the DOF is considerably less with the IAT than with the IVT, less than 2 mm if the entire aperture is used. Since the DOF seen in Fig. 10 ▶ is larger (>5 mm), we conclude that other factors are limiting the measured focal spot size (high-frequency instabilities in the IAT and/or measurement knife-edge mounting). The theoretical focal spot size, based on the measured source size, should be below 300 nm.

#### 3.4.2. IAT and IVT as a compound transfocator

The two transfocators have been used in series to form a compound focusing device. By using the IVT to collimate/pre-focus the beam, enormous gains in flux can be achieved with only slight degradation of the spot size. Such an application has been demonstrated at 29.6 keV.

By correcting the focal length of the IAT, it is possible to recover an only slightly broadened spot, with gains of more than 30 and a beam broadening of only about 75%. The most advantageous arrangement seems to be with the IVT focusing just downstream from the IAT; when the image distances of the two transfocators are close there is significant broadening (approximately a factor of three after optimization). The maximum flux is achieved when the image distance of the IVT is near that of the IAT (the IAT is within the DOF of the IVT), but a higher gain (owing to smaller spot size) is achieved when focusing the IVT downstream from the IAT.

The approximate change in focal length of the IAT used in conjunction with 11 Be lenses in the IVT can be estimated using the thin-lens approximation (Hecht, 1974 ▶). The back focal length (BFL) with respect to the IAT is
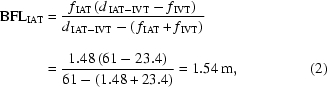
where *f*_IAT_ = 1.48 m and *f*_IVT_ = 23.4 m are the focal lengths of IAT and IVT, respectively, and *d*_IAT–IVT_ = 61 m is the distance between the transfocators. The new image distance for the compound transfocator is then
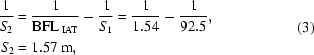
where *S*_1_ and *S*_2_ are the object and calculated image distances for the compound transfocator relative to the IAT, respectively.

This very approximate calculation gives a surprisingly accurate result, as the change in image distance *S*_2_ (= 1.5 m with only the IAT) is found to be about 55 mm (as opposed to 70 mm as predicted here). With 12 lenses, the result of the calculation is essentially identical (the BFL changes only in the third decimal place), although the change in *S*_2_ is found to be closer to 90 mm; clearly the thickness of the lenses should be taken into account. More complete calculations are underway.

As the absorption of the Be lenses in the IVT is negligible, use of the IVT therefore allows focusing of almost the entire beam into a microfocused spot. By varying the focal length of the IVT, different defocusing/flux ratios can be selected. For example, we observe a 20× increase in flux when the spot is defocused by about 50%, and a 30× increase when the spot is defocused by a factor of two.

#### 3.4.3. Use of IVT with nanolens chip

**Figure 11:**
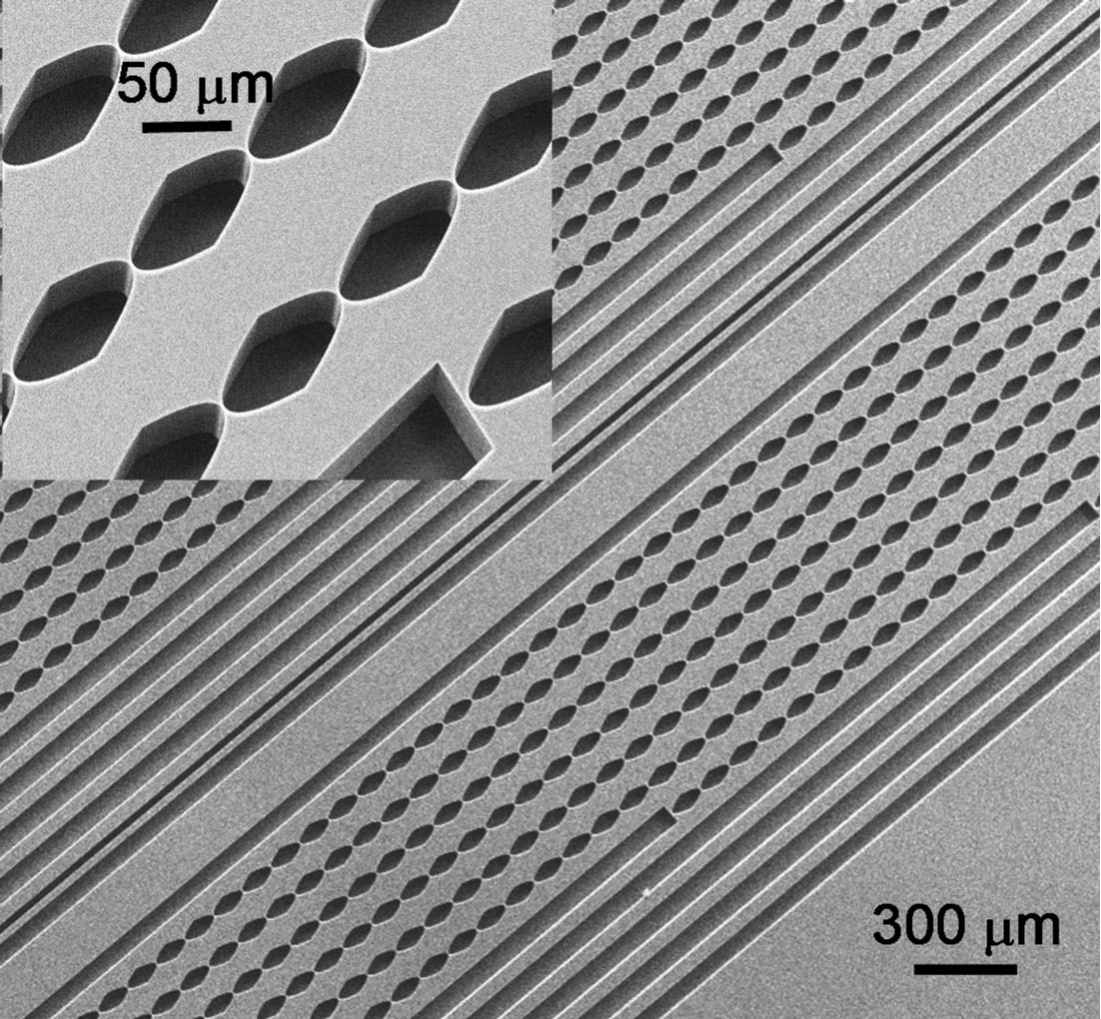
Scanning electron microscope image of a nanofocusing lens chip and individual lenses (insert).

The IVT can also be used to great effect with nanofocusing optics such as the Si nanofocus chip (Snigirev *et al.*, 2007 ▶, 2009*b* ▶) used for these purposes at ID11. The nanofocusing lens chip comprises ten lens arrays optimized for the energy range from 10 to 55 keV with a step of 5 keV; at these exact energies the focal distance is 10 cm. Other energies can be used by varying the focal distance. The length of the individual lenses is 100 µm and their aperture and depth are both 50 µm. The web thickness of individual lenses on the optical axis is 2 µm and the surface roughness is of the order of 20 nm. A scanning electron microscope image of nanolenses is shown in Fig. 11 ▶. For 57.2 keV focusing we used a lens consisting of 80 individual lenses with a focal distance of about 30 cm.

As the focal spot of the IVT at 92 m is slightly smaller than the physical aperture of this device (50 µm), essentially all of the vertical beam can be refocused. By adjusting the focal length of the two lens systems, a factor of 35 in flux was gained while the 57.2 keV spot was broadened from about 180 nm to about 450 nm.

## 4. Discussion and conclusion

We have introduced an X-ray transfocator based on compound refractive lenses as a very flexible and powerful focusing/collimating device. Used alone or in conjunction with other optical elements the IVT can provide from moderate to nanofocused beams over a very large energy range. When used as a pre-focusing condenser, truly spectacular gains in flux can be achieved, with only moderate effect on focal size. Used alone or with a non-focusing monochromator, the IVT provides significant gains in flux, particularly on long beamlines. A further interesting characteristic of the purely chromatic focusing of compound refractive lenses is that used alone or with other monochromators they provide harmonic suppression, unlike typical periodic monochromators such as crystals and multilayers, in which higher-order reflections diffract multiples of the fundamental energy.

The IVT is trivial to align and extremely stable. It can be easily removed and reproducibly re-inserted in the beam when variable beam size is needed. Unlike diffractive and reflective optics which multiply angular instabilities, refractive optics are, like zone plates, entirely insensitive to vibrations which cause angular changes, the predominant vibrational modes of the mounting. This leads to a very high stability, critical for microfocusing and nanofocusing applications. In particular, using the IVT and our double Laue monochromator (Vaughan, 2010 ▶), we were able to image a vertical source of 21 µm, close to the true source size and among the smallest observed in a monochromatic beam. This high stability also means that the IVT can be used to characterize the source. At ID11’s end-station 94 m from the source the focusing ratio is about 1:2, which means the source is focused and can be measured with a normal high-resolution detector, allowing rapid source characterization (up to 1 kHz with a fast camera) and the measurement of submicroradian angular source size.

One or more transfocators are now used for the vast majority of experiments at ID11, whether powder, single-crystal or polycrystal diffraction experiments. Furthermore, permanent implantation of the devices in-line allows the beamline to function as an X-ray analogue to an electron microscope, by switching between imaging and diffraction modes while preserving the beam path. Coupled with the parallel detector schemes employed at ID11 (Vaughan, 2010 ▶; Poulsen *et al.*, 2010 ▶), this allows for samples to be studied by different methods simultaneously or almost simultaneously.

For example, by tuning the setting of the transfocators, a wide variety of experimental conditions may be created. The beam can be focused, pre-focused or collimated for high-resolution applications such as peak-shape analysis (Ungar *et al.*, 2010 ▶; Nisr *et al.*, 2011 ▶) or for post-monochromating with a high-resolution monochromator. By focusing on the detector or beam stop, small-angle scattering may be measured, even at high energy (Byelov *et al.*, 2010 ▶; Bosak *et al.*, 2010 ▶). Finally, by adjusting the sample position with respect to the optics, the beamline can also perform imaging as a high-resolution full-field microscope (Schroer *et al.*, 2001 ▶; Vaughan, 2010 ▶) with the IVT as a condenser and other compound refractive lenses as an objective. The length of the beamline allows for very high magnification ratios in such a configuration. For example, with the IVT collimating a beam onto a sample at 42 m from the source, a second series of lenses just behind the sample can act as a focusing element to imaging onto a detector 99 m from the source, giving a magnification of more than of 100× or more, and allowing resolutions of better that 100 nm (Vaughan, 2010 ▶). Such an arrangement allows simple changing between absorption and phase-contrast modes, as well as diffraction and imaging modes.

## Figures and Tables

**Figure 7:**
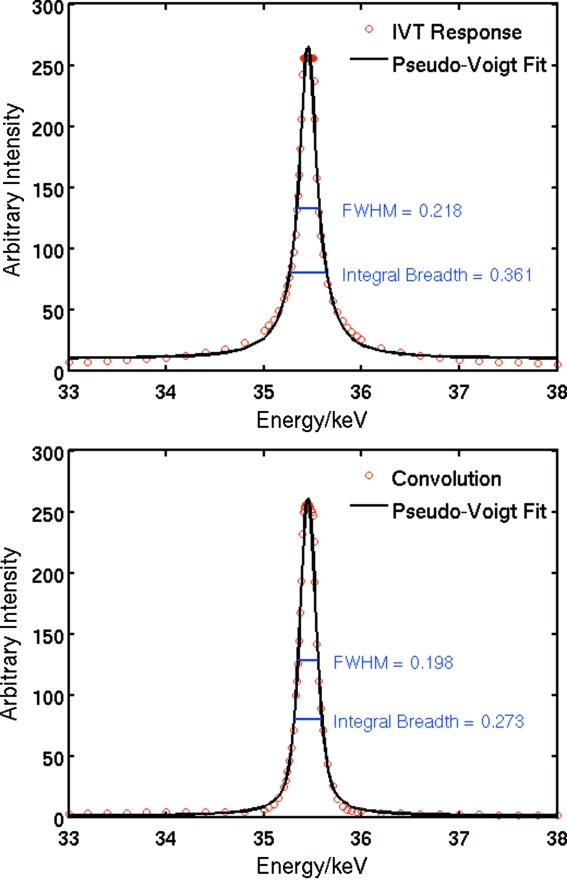
Pseudo-Voigt fits to a theoretical IVT response curve (top), and the response curve convoluted with a U22 undulator peak (bottom). The latter has greatly reduced tails and a better signal-to-noise ratio.
